# Brain Stimulation and Neuroplasticity

**DOI:** 10.3390/brainsci11070873

**Published:** 2021-06-30

**Authors:** Ulrich Palm, Moussa A. Chalah, Samar S. Ayache

**Affiliations:** 1 Department of Psychiatry and Psychotherapy, Klinikum der Universität München, 80336 Munich, Germany; 2 Medical Park Chiemseeblick, Rasthausstr. 25, 83233 Bernau-Felden, Germany; 3 EA4391 Excitabilité Nerveuse & Thérapeutique, Université Paris Est Créteil, 94010 Créteil, France; moussachalah@gmail.com (M.A.C.); samarayache@gmail.com (S.S.A.); 4 Service de Physiologie—Explorations Fonctionnelles, Hôpital Henri Mondor, Assistance Publique—Hôpitaux de Paris, 94010 Créteil, France

Electrical or magnetic stimulation methods for brain or nerve modulation have been widely known for centuries, beginning with the Atlantic torpedo fish for the treatment of headaches in ancient Greece, followed by Luigi Galvani’s experiments with frog legs in baroque Italy, and leading to the interventional use of brain stimulation methods across Europe in the 19th century. However, actual research focusing on the development of transcranial magnetic stimulation (TMS) is beginning in the 1980s and transcranial electrical brain stimulation methods, such as transcranial direct current stimulation (tDCS), transcranial alternating current stimulation (tACS), and transcranial random noise stimulation (tRNS), are investigated from around the year 2000.

Today, electrical, or magnetic stimulation methods are used for either the diagnosis or exploration of neurophysiology and neuroplasticity functions, or as a therapeutic intervention in neurologic or psychiatric disorders (i.e., structural damage or functional impairment of central or peripheral nerve function).

This Special Issue ‘Brain Stimulation and Neuroplasticity’ gathers ten research articles and two review articles on various magnetic and electrical brain stimulation methods in healthy populations and in patients with neurologic or psychiatric disorders. Articles were clustered to either belong to the magnetic or electrical stimulation techniques.

Transcranial magnetic stimulation was used by Haeckert et al. [1] to assess neurophysiologic effects in healthy volunteers. They investigated the aftereffects of continuous theta burst stimulation (cTBS) with 300 and 600 pulses and found no relevant changes of motor evoked potentials (MEP) during an observation period of 30 min. This study does not support the findings of some studies reporting that cTBS 300 increases and cTBS 600 decreases MEP in relaxed healthy volunteers. It adds evidence to the broad variability, i.e., the lack of a clear direction of MEP changes after cTBS, which has also been shown in other studies.

Hoonhorst et al. [2] used TMS pulses to detect the central motor conduction time (CMCT) in patients following an ischemic stroke. Therefore, the stimulation was applied over the non-infarcted hemisphere, particularly over the primary motor cortex, to generate MEP. They showed that CMCT was prolonged directly after a stroke in 60% of patients and did not normalize within 11 days. Although the mechanism for this phenomenon remains unclear, the authors not only suggested the contribution of transcallosal, but also reticulospinal, tectospinal, and rubrospinal pathways at its basis.

Repetitive peripheral magnetic stimulation (rPMS) with 25 Hz frequency was used by Malejko et al. [3] and revealed higher pain thresholds in patients with borderline personality disorder (BPD) compared to healthy controls and patients with major depression. Furthermore, patients with BPD did not show a modulation in their emotional reaction to increasing intensity levels of unpleasant somatosensory stimulation. Study results suggest an altered pain processing in BPD and are in line with previous studies.

Finally, in a review article, Klimek and Rogalska [4] elucidate the role of extremely low-frequency magnetic fields (ELF-MF) on human health. These magnetic fields may be caused naturally (e.g., solar activity), or by humans (e.g., electronic devices, transmission lines). Principally, magnetic fields can influence hormones, neurotransmission, inflammation, and cellular signal cascades. The authors reviewed the literature of the last decade dealing with the consequences of magnetic field exposure in daily life and found that ELF-MF may cause both beneficial and detrimental stress to cellular functioning. Due to a mass of confounding factors, a clear distinction of a detrimental threshold is not possible at this stage and standardized measurements are needed for future studies.

Transcranial direct current stimulation (tDCS) was used by Adam et al. [5] in a randomized study to investigate the effects on serum mature Brain Derived Neurotrophic Factor (mBDNF) in patients with schizophrenia and auditory verbal hallucinations. Interestingly, a single session of active left-side prefrontal-temporoparietal stimulation decreased mBDNF levels compared to sham tDCS, suggesting a potential modulation of mBDNF- tropomyosin receptor kinase B pathways in order to promote neuroplasticity in the central nervous system. However, the role of BDNF in tDCS-elicited neuroplasticity remains unclear.

Another study using tDCS investigated the effects of visual cortex stimulation in patients with proliferative diabetic retinopathy [6]. De Venecia and Fresnoza showed that cathodal stimulation decreased reaction time and improved visual acuity, whereas sham stimulation had no effect. The authors suggest that there is an improvement in visual discrimination after reduction of neuronal noise by cathodal stimulation.

The treatment of Parkinson’s Disease Related Fatigue (PDRF) with tDCS is proposed by Zaehle in a review article [7]. He showed that PDRF is largely overlooked in the clinical management of Parkinson’s Disease and severely impacts the quality of life in these patients. PDRF shows correlation with the symptoms of depression, therefore an anodal stimulation of left prefrontal cortical areas analogously to the treatment of depression is suggested.

A second article dealing with Parkinson’s disease evaluated the long-term course of nine patients receiving extradural motor cortex stimulation (EMCS). Piano et al. [8] found that treatment was safe and there was a slight improvement of motor fluctuations and dyskinesias, also reflected by an improvement in the quality of life.

Chen et al. [9] reported the differential effects of 10 Hz and 20 Hz tACS on cerebral activation patterns in patients with chronic stroke. Data acquisition by functional Magnetic Resonance Imaging (fMRI) showed that 20 Hz tACS might facilitate local segregation in motor-related regions and global integration at the whole-brain level. Furthermore, 20 Hz, but not 10 Hz tACS, increased nodal clustering. The authors suggest that 20 Hz tACS might induce higher modulation effects, which could be used in rehabilitation therapies to facilitate neuromodulation.

Home treatment with tACS to improve migraine attacks was proposed by Antal et al. [10]. Patients were trained to perform a visual cortex stimulation when a migraine attack started. If the attack did not resolve within two hours after stimulation, patients were allowed to take their rescue medication. It was calculated that 21% of migraine attacks were terminated by active tACS, compared to 0% in the sham group. The authors suggest that the inhibitory character of 140 Hz tACS could reduce neuronal activity during the occurrence of migraines.

Kim et al. [11] reported the use of electroacupuncture (4 points) in combination with computer-based cognitive rehabilitation (CCR) to improve mild cognitive impairment. Compared to a control group receiving CCR only, electroacupuncture and CCR showed no superiority in terms of cognitive improvement, which was seen as a CCR effect in both groups.

Finally, Ko et al. [12] investigated the effects of noisy galvanic stimulation (GVS) of the mastoid processes in patients with bilateral vestibular hypofunction and in healthy volunteers. They found an improvement of sway in both groups during walking and standing, and an increase in alpha, beta, gamma, and theta band power in the left parietal lobe in both groups. It is postulated that GVS can improve postural stability in patients with vestibular hypofunction.

In summary, the articles in this Special Issue cover a broad range of clinical applications of different (non)invasive stimulation techniques for modulating various disorders or for neurophysiological investigations. Of note, the actual literature presents some limitations related to the methodological differences, the scarcity of studies or the small sample size. This reflects the need for further large-scale studies in the emerging field of novel brain stimulation techniques.
